# A Synaptically Connected Hypothalamic Magnocellular Vasopressin-Locus Coeruleus Neuronal Circuit and Its Plasticity in Response to Emotional and Physiological Stress

**DOI:** 10.3389/fnins.2019.00196

**Published:** 2019-03-20

**Authors:** Oscar R. Hernández-Pérez, Vito S. Hernández, Alicia T. Nava-Kopp, Rafael A. Barrio, Mohsen Seifi, Jerome D. Swinny, Lee E. Eiden, Limei Zhang

**Affiliations:** ^1^Departamento de Fisiología, Facultad de Medicina, Universidad Nacional Autónoma de México, Mexico City, Mexico; ^2^Instituto de Física, Universidad Nacional Autónoma de México, Mexico City, Mexico; ^3^School of Pharmacy and Biomedical Sciences, Institute for Biomedical and Biomolecular Science, University of Portsmouth, Portsmouth, United Kingdom; ^4^Section on Molecular Neuroscience, National Institute of Mental Health-IRP, Bethesda, MD, United States

**Keywords:** arginine vasopressin, RNAscope, Fluoro-Gold, Morris water maze, electron microscopy

## Abstract

The locus coeruleus (LC)-norepinephrine (NE) system modulates a range of salient brain functions, including memory and response to stress. The LC-NE system is regulated by neurochemically diverse inputs, including a range of neuropeptides such as arginine-vasopressin (AVP). Whilst the origins of many of these LC inputs, their synaptic connectivity with LC neurons, and their contribution to LC-mediated brain functions, have been well characterized, this is not the case for the AVP-LC system. Therefore, our aims were to define the types of synapses formed by AVP+ fibers with LC neurons using immunohistochemistry together with confocal and transmission electron microscopy (TEM), the origins of such inputs, using retrograde tracers, and the plasticity of the LC AVP system in response to stress and spatial learning, using the maternal separation (MS) and Morris water maze (MWM) paradigms, respectively, in rat. Confocal microscopy revealed that AVP+ fibers contacting tyrosine hydroxylase (TH)+ LC neurons were also immunopositive for vesicular glutamate transporter 2, a marker of presynaptic glutamatergic axons. TEM confirmed that AVP+ axons formed Gray type I (asymmetric) synapses with TH+ dendrites thus confirming excitatory synaptic connections between these systems. Retrograde tracing revealed that these LC AVP+ fibers originate from hypothalamic vasopressinergic magnocellular neurosecretory neurons (AVPMNNs). MS induced a significant increase in the density of LC AVP+ fibers. Finally, AVPMNN circuit upregulation by water-deprivation improved MWM performance while increased Fos expression was found in LC and efferent regions such as hippocampus and prefrontal cortex, suggesting that AVPMMN projections to LC could integrate homeostatic responses modifying neuroplasticity.

## Introduction

Converging pharmacological, physiological and behavioral data indicate that the neurohormone arginine vasopressin (AVP) system is principally linked to the homeostatic regulation of fluid balance (Armstrong, 2004), yet ascending vasopressinergic projections that connect the physiology of water regulation with emotional context and adaptive behaviors have been reported (de Wied et al., 1993; Hernandez et al., 2012; Zhang et al., 2012, 2016, 2018; Zhang and Eiden, 2018). Historically, the actions of AVP within the brain were considered to primarily occur in a paracrine manner, for example, via dendritic release from hypothalamic neurons and further diffusion to target brain regions via the cerebrospinal or interstitial fluids (Leng et al., 1992; Landgraf and Neumann, 2004; Ludwig and Leng, 2006). However, we and others have since demonstrated direct synaptic connections between vasopressinergic magnocellular neurosecretory neurons (AVPMNNs) of the hypothalamus and other brain regions including hippocampus (Cui et al., 2013; Zhang and Hernandez, 2013), amygdala (Hernandez et al., 2016), and lateral habenula (Zhang et al., 2016, 2018) via dual AVPMNN projections to these regions and to the neurohypophysis (Hernandez et al., 2015). This raises the possibility of direct AVPMNN projections to additional brain nuclei that participate in integrating homeostatic with behavioral regulation (Zhang and Eiden, 2018). AVP-immunopositive (AVP+) axons have indeed been identified in a myriad of brain regions involved in such functions, including the pontine nucleus *locus coeruleus* (LC) (Buijs, 1978; Rood and De Vries, 2011). However, the source of these inputs, and therefore the putative regulatory circuits in which they participate, have not been identified.

The LC (also called nucleus pigmentosus ponti) are bilateral dense groups of cells located in the pontine tegmentum, specifically in the lateral-rostral part of the floor of the 4th ventricle. LC neurons are identified by their expression of the norepinephrine synthesizing enzymes tyrosine hydroxylase (TH) and dopamine-beta-hydroxylase (DBH), but not phenylethanolamine N-methyltransferase, thereby confirming their principal neurochemical signature of norepinephrine (NE) (Kobayashi et al., 1974; Swanson, 1976; Levitt and Moore, 1979). LC neurons provide the major source of NE throughout most of the brain (Robertson et al., 2013; Schwarz and Luo, 2015). The LC-NE system modulates some of the most salient brain functions, such as arousal, learning and memory and the cognitive response to stress (Berridge and Waterhouse, 2003; Atzori et al., 2016). At the synaptic level, NE facilitates synaptic plasticity by recruiting and modifying multiple molecular elements of synaptic signaling, including specific transmitter receptors, intracellular protein kinases, and translation initiation (Maity et al., 2015; Nguyen and Gelinas, 2018). All such LC-NE functions are strongly aligned with the levels of LC neuronal activity. While LC neurons are spontaneously active, their firing rates are strongly influenced by their afferent inputs, many of which contain an array of neuropeptides (Palkovits and Brownstein, 1983), including corticotropin-releasing factor (CRF) (Swinny and Valentino, 2006; Swinny et al., 2010) and AVP (Buijs, 1978). Regarding the former, there is consensus that CRF fibers in LC are of hypothalamic (PVN parvocellular) origin (Valentino and Van Bockstaele, 2008). While a large body of data demonstrate the origins of CRF and other LC afferents (Schwarz and Luo, 2015), the precise source of AVP+ axons in the LC has yet to be identified, even though hypothalamic paraventricular and supraoptic regions are known sources for afferents to LC (Schwarz et al., 2015). Furthermore, conclusive evidence for AVP+ fibers making synaptic contact with LC neurons has yet to be reported.

We recently reported on the molecular and physiological correlates of the AVP-receptor system in the mouse LC (Campos-Lira et al., 2018). In the current study, we expand upon these data to demonstrate that AVP+ axons make excitatory synaptic contact with TH neurons, at the ultrastructural level, and that these axons originate from discrete hypothalamic nuclei, thereby identifying specific AVP hypothalamic-LC circuits. We further demonstrate the potential engagement of these circuits in response to life experiences which require the homeostatic properties of both the LC and the hypothalamus.

## Materials and Methods

### Animals

Wistar rats from a local animal breeding facility were used throughout this study. All procedures were approved by the Research and Ethics Committee of the Faculty of Medicine, Universidad Nacional Autónoma de México (CIEFM 062/2016). Animals were housed three per cage under controlled temperature (22°C) and illumination (12 h), with water and food *ad libitum*. After surgery, animals were kept warm until fully recovered from anesthesia and then kept individually under the above-mentioned conditions for 1 week and returned to the original housing conditions.

### Immunohistochemistry (IHC) for Light and Transmission Electron Microscopy (LM and TEM)

Male rats of 270 ± 30 g were deeply anesthetized with an overdose of sodium pentobarbital (63 mg/kg, Sedalpharma, Mexico) and perfused transaortically with 0.9% saline followed by cold fixative containing 4% of paraformaldehyde in 0.1 M sodium phosphate buffer (PB, pH 7.4) plus 15% v/v saturated picric acid for 15 min (0.05% of glutaraldehyde was added to fixative for the samples intended for TEM). Brains were immediately removed, blocked, then thoroughly rinsed with 0.1 M PB until they were clear of fixative. The brains were then sectioned using a Leica VT 1000S vibratome, at 70 μm thickness in the horizontal plane.

**FIGURE 1:**
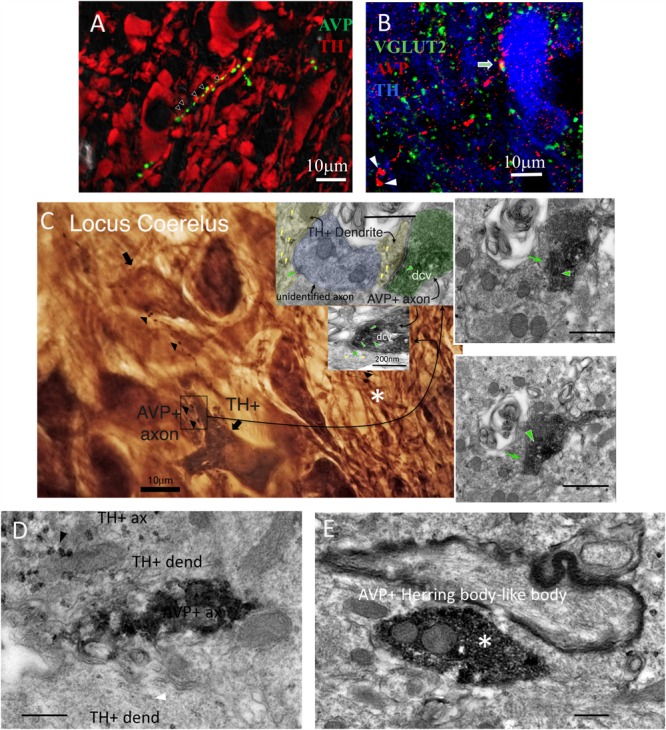
AVP–VGLUT2 immunopositive (AVP+/VGLUT2+) fibers establish Gray type I (asymmetric) synapses onto tyrosine hydroxylase immunopositive (TH+) dendrites within the LC. **(A,B)** Confocal photomicrographs showing AVP+ fibers making contacts with TH+ dendrites **(A)** and the co-localization of AVP+/VGLUT2+ segments (**B**, two Herring body-like bodies were indicated by arrow heads). **(A)** AVP reaction was made using the antibody gifted from H. Gainer laboratory and image was taken at 0.5 AU (Airy Units, using Leica SP5 confocal system) optical section thickness of around 0.596 μm. This measure helps to improve the optical resolution to visualize the contact points in a strongly labeled tissue. The panel **B** was from mouse LC tissue using a different antibody (Abcam ab39363) and taken at 1 AU (optical section thickness was around 0.892 μm). **(C)** Photomicrograph taken from a pre-embedding immunoreaction of trimmed resin capsule surface (the “pyramid,” *osmicated* losing the purple color under LM), containing LC prepared for electron microcopy (EM), using DAB/VIP (Very Intense Purple) double peroxidase-chromogen immunostaining for electron microscopy. AVP+ fibers were evidently making contact with TH+ dendritic segments indicated by arrowheads. The inserts are TEM micrographs from serial samples of the region indicated by rectangle area in **C**. The image shows an AVP+ axon with a terminal (depicted in four serial sections) containing AVP+ dense-core vesicles (dcv, indicated with green arrowhead), establishing a Gray-type I synapse onto a TH+ dendrite (TH is demonstrated by granular labeling produced by VIP reagent at electron microscopy level, yellow arrowheads). Postsynaptic density (PSD), a TEM feature of a Gray type I synapse, which is generally indicative of a glutamatergic synapse, are indicated by green arrows. **(D)** TEM micrograph showing one segment of the same AVP+ profile (DAB-nickel labeling) coursing in parallel with two TH+ (VIP labeling) dendrites. Note that the VIP labeling for dendrite (white arrowhead) was weaker than for axonal segment (black arrowhead). **(E)** An Herring body-like body (large axonal varicosity), an anatomical feature of the vasopressinergic magnocellular neurosecretory neurons (white asterisks of panel **B,E**) examined under EM. Scale bars: 400 nm unless stated otherwise.

For LM IHC, non-specific binding of the secondary antibodies was minimized by incubating sections containing LC with 20% normal donkey serum (NDS) in Tris-buffered (0.05 M, pH 7.4) saline (0.9%) plus 0.3% of Triton X-100 (TBST) for 1 h at room temperature (RT). The sections were then incubated with the following primary antibodies: rabbit anti-AVP antibody (kind gift of Dr. Ruud M. Buijs; Buijs et al., 1989), mouse anti-AVP antibody (kind gift of Dr. Hal Gainer; Alstein et al., 1988), rabbit anti-AVP (Abcam, ab39363, for Figure 1B) sheep anti-tyrosine hydroxylase (TH) [Abcam, ab113; guinea pig anti-VGLUT2 (Frontier Institute, VGLUT2, GP-Af810)]. The next day, the sections were washed with TBST for 30 min after which they were incubated at RT in a cocktail of an appropriate mixture of secondary antibodies, conjugated with Alexa Fluor 488, Alexa 594 and indocarbocyanine (Cy5), all provided by Jackson ImmunoResearch, for 2 h. The sections were washed in TBST and were mounted on glass slides, air dried and coverslipped using Vectashield mounting medium (H-1000, Vector Laboratories Inc.).

For double peroxidase-chromogen immunostaining and electron microscopy (TEM), brain sections containing LC, were cryoprotected with 10% and then 20% sucrose in PB (under gentle agitation until the sections sank). Permeability of the tissue was then enhanced by rapidly freezing and thawing sections using liquid nitrogen. Sections were then thoroughly rinsed with PB 0.1 M and non-specific secondary antibody binding was minimized with 20% NDS in TBS for 1 h at RT. The sections were then incubated with rabbit anti-AVP and sheep anti-TH antibodies (see above) in TBS plus 1% NDS for 48 h at 4°C with gentle agitation. After rinsing in TBS, incubation was continued with first secondary antibody swine anti-rabbit IgG conjugated with horseradish peroxidase (HRP) (1:100, Dako P021702, Copenhagen, Denmark), in TBS containing 1% of NDS, overnight at 4°C. Sections were then rinsed and peroxidase enzyme reaction was carried out using the chromogen 3,3′-diaminobenzidine (DAB, 0.05%, Electron Microscopy Sciences) and hydrogen peroxide (H_2_O_2_, 0.01%) as the substrate. The reaction end product in some sections was intensified with nickel. Subsequently, sections were incubated with 2nd secondary antibody, biotinylated goat anti sheep antibody (Jackson ImmunoResearch Laboratories) and then incubated with Vectastain standard ABC kit [VECTASTAIN^®^Elite^®^ABC HRP Kit (Peroxidase, Standard), Cat. No: PK-6100, Vector Laboratories, Burlingame, CA, United States]. The TH immunoreactivity was then visualized by using a Vector-VIP (*very intense purple*) peroxidase substrate kit [VECTOR^®^VIP Peroxidase (HRP) Substrate Kit; Vector Laboratories]. This procedure yields a reaction product that appears purple in the light microscope and granular or particulate in the electron microscope. Sections were then post-fixed with 1% osmium tetroxide in 0.1 M PB for 1 h and dehydrated through a series of graded alcohols (including 45 min of incubation in 1% uranyl acetate in 70% ethanol), then transferred to propylene oxide, followed by Durcupan ACM epoxy resin (Cat. No. 100503-434, Electron Microscopy Sciences). Sections were flat embedded on glass microscope slides, and the resin was polymerized at 60°C for 2 days. After removing the coverslip, LC containing regions, identified by TH immunoreactivity, were sectioned and carefully re-embedded in capsules in Durcupan resin. Ultrathin serial sections (70 nm) were prepared with an ultramicrotome, collected on pioloform-coated single slot grids and examined with a Philips CM100 transmission electron microscope. Digital electron micrographs were obtained with a digital micrograph 3.4 camera (Gatan Inc., Pleasanton, CA, United States).

### RNAscope ISH Assays

Rats were deeply anesthetized and decapitated using a small animal guillotine (Kent Scientific corp.). Brains were removed and rapidly frozen using dry-ice powder. The fresh-frozen tissue was sectioned (12 μm thick) using a cryostat (Leica CM-1520) and mounted on positively charged glass slides (Fisher Scientific, Pittsburgh, PA, United States). The RNA probes for *in situ* hybridization used in this study to identify the genes of tyrosine hydroxylase (TH), V1a (Avpr1a), and V1b (Avpr1b) receptors were designed and provided by Advanced Cell Diagnostics (Hayward, CA, United States). All staining steps were performed following RNAscope^®^2.5 HD Duplex Assay protocol for fresh frozen sections.

### Fluoro-Gold Retrograde Tracing

The FG retrograde injection method was according to previously described protocols (Schmued and Fallon, 1986; Morales and Wang, 2002; Yamaguchi et al., 2011; Zhang and Hernandez, 2013). A total of twenty 300g Wistar male rats were used in this experiment. Rats were anesthetized with xylazine (Procin, Mexico) (20 mg/ml) and ketamine (Inoketam, Virbac, Mexico) (100 mg/ml) in a 1:1 volume ratio and administered intraperitoneally (*i.p.)* at a dose of 1 ml/kg of body weight. Deeply anesthetized rats were placed in a stereotaxic apparatus and the retrograde tracer Fluoro-Gold (FG, Fluorochrome, LLC, Denver, Colorado 80218, United States), dissolved 1% in 0.1 M cacodylate buffer (pH 7.5), was delivered iontophoretically (Value Kation Sci VAB-500) at the following coordinates: Bregma -9.48 mm, lateral 1.20 mm, and dorso-ventral 7.30 mm, via a glass micropipette (inner tip diameter of approximately 20 μm, current pulses of 0.1 μA, at 0.2 Hz, with a 50% duty cycle) for 20 min. The micropipette was left in place for an additional 10 min to prevent backflow of the tracer up the injection track after each injection. Upon recovery, the rats were administered 0.4 mg/kg *i.p.* Ketorolac (Apotex, Mexico) and 50 mg/kg *i.p.* ceftriaxone (Kendric, Mexico) as analgesic/anti-inflammatory and antibiotic agents, respectively. One week after the FG injections, the rats were perfused and brains sectioned in the coronal plane. Injection sites were evaluated and the inclusion criteria include: (1) the center of the injection was within the 300 μm of the core of LC and (2) there was no visible cerebro-spinal-fluid (CSF) leaking of FG, evidenced by diffused and bilateral signals. The selected cases were further processed for IHC against TH in the brain stem and AVP in the anterior hypothalamus sections.

### Maternal Separation (MS) Protocol

The MS (3 h daily, MS 3 h) procedure is described in detail elsewhere (Zhang et al., 2012). Briefly, female and male adult rats were mated for 2 days. During the last week of gestation, female rats were single-housed in standard rat Plexiglas cages and maintained under standard laboratory conditions. On the day after parturition, postnatal day (PND) 2, each litter was culled to 7–8 pups, of which 5–6 were males. During the period from PND 2–PND 16, the pups were separated daily from their dams, and placed into an incubator at 29°C ± 1°C, between 09:00 and 12:00 h. After this period rats were returned to their home cages. After ending the MS protocol, animals were left undisturbed until the weaning at PND 28, when male and female rats were separated. Bedding was changed twice a week, with minimum disturbance. At PND 75, male rats of 270 ± 30 g were not allowed access to water for 24 h (WD24h) before perfusion-fixation, according to the above-mentioned protocols, in order to minimize variability in basal AVP immunoreactivity, as demonstrated previously (Verney, 1947; Fitzsimons and O’Connor, 1976; Robertson et al., 1976; Zhang et al., 2010), thereby enhancing comparability of subjects. Following fixation, tissue sections were prepared and immunoreactivity for AVP within the LC was carried out as above described.

### Optical Density Analysis

For optical density analysis of IHC, the area occupied by the signal was calculated in 10 fields in control and MS animals using a modification of the protocol described elsewhere (Ying et al., 2017). Briefly, all images were acquired under identical conditions. Using ImageJ, images were converted to grayscale. Thresholds were manually set until the majority of the area occupied by the labeled fibers was highlighted and maximally separated from the background noise. Images were converted to binary format and the threshold areas were measured. The results were expressed as mean ± SEM percentage of control and compared with a Student *t*-test.

### Morris Water Maze Test

The Morris water maze (MWM) (Morris, 1984) assesses spatial learning and memory retention. The experimental design we used consisted of a black circular pool (diameter: 156 cm; height: 80 cm) filled with water to a height of 30 cm and temperature of 25 ± 1°C, with visual cues placed on the wall of the pool. Four virtual quadrants were named I, II, III, IV (see Figure 5 panes C and D for a graphical description). A circular black escape platform (diameter 12 cm) was submerged 1 cm below the water surface to serve as an escape platform. The tests were video recorded under dim red light. This setting has proved to be useful for spatial learning and memory retention assessments as previously described (Zhang et al., 2008; Hernandez et al., 2012).

Ten rats participated in this test. Animals were kept in a light-dark cycle 12:12, with light on at 7:00 am of solar time, which is denominated as the *zeitgeber* time 0, *i.e.*, circadian time 0 (CT0) and were separated in two groups: control with food and water *ad libitum* and water deprivation 24 h group (WD24h). The test was performed during rat inactivity period aiming to avoid the spontaneous high Fos expression in LC during the early activity period. Two assessments were made sequentially: the “morning” test (MT: performed between CT3–CT5) and the “afternoon” test (AT: performed between CT8–CT10). Before the MT, a pre-test habituation was done, which consisted in placing subjects from one group into the pool for 2 min to allow them to habituate the water. Rats are natural swimmers. Hence, this procedure reduces the possible stress of being immersed into water during the tests as well as to activate the innate swimming ability while making them acquainted to the environment.

The MT of MWM started by placing the escape platform in the quadrant II. Each subject was carefully introduced into the pool starting at quadrant I. Time elapsed between this event and subject arrival at the escape platform was recorded. In the first trial, subjects were allowed to explore the entire pool for 60 s, then the experimenter guided them to the platform, allowing them to climb onto the platform and to observe the location and environment for 5 s (see Figure 5, panels C and D, symbolized with dashed lines for a graphical description).

Six trials were completed for each subject, with intervals of 5 min between each of them. The rats were returned to the resting room with water and food *ad libitum* for 3 h. Then the AT started at CT8 repeating the same procedure modifying the location of the escape platform to the quadrant I and the starting point to the quadrant III.

### Fos IHC and Assessment

To evaluate the neuronal activity of LC, hippocampus and prefrontal cortex, the protein product of the proto-oncogene Fos was used. Since Fos is best detected in the interval between 60 and 120 min after a neuron is activated (Kovacs, 1998, 2008), we perfused the rats 90 min post-AT (MWM). For IHC, every third section containing the brain nuclei was blocked with 10% NDS in Tris-buffered (0.05M, pH 7.4) saline (0.9%) plus 0.3% of Triton X-100 (TBST) for 1 h at RT and then immunoreacted overnight with rabbit anti-Fos primary antibody (SC52, 1:1000, Santa Cruz Biotechnology, Santa Cruz, CA, United States) in TBST + 1% NDS at 4°C with gentle shaking. Afterward, sections were rinsed three times for 10 min with TBST and then incubated for 2 h at RT with biotinylated goat anti-rabbit secondary antibody (1:200; Vector Labs, Burlingame, CA, United States). Finally, sections were incubated in avidin-biotin-peroxidase complex (Elite ABC Kit, Vector Labs) for 1 h at RT. Peroxidase was detected using diaminobenzidine 0.05% as chromogen. Sections were rinsed and permanently mounted with Permount mounting medium (Electron Microscopy Sciences, Hatfield, PA, United States). Fos immunoreactive nuclei per 0.0346 mm^2^ (area of a visual field: VF through the 100× objective) were counted using a Nikon Eclipse 50i microscope. The results were expressed as mean ± SEM nuclei/VF and compared with a Student *t*-test.

## Results

### Synaptic Connectivity Between AVP+ Axons and LC Neurons

In mouse, sparse AVP+ axons have been reported in the region occupied by LC, using single labeling light microscopy (Rood and De Vries, 2011). We recently expanded upon these data to demonstrate that AVP+ axons in the mouse LC are located in close apposition to inhibitory and excitatory synaptic marker proteins (Campos-Lira et al., 2018). However, it is unclear whether the same association of AVP with synaptic molecular machinery exists in other species such as the rat, and whether the signal is located within synaptic junctions, or on neighboring compartments. We therefore began by examining the association of AVP+ profiles with such molecular signatures of synaptic transmission, in rat LC at the light microscopical level.

Using widely validated AVP antibodies (rabbit and mouse anti AVP), generous gifts from Ruud Buijs and Harold Gainer, respectively, we detected sparse AVP+ fibers in the LC which were closely apposed to tyrosine hydrolase (TH) immunoreactive dendrites (Figure 1A) and somata (Figure 1B). In contrast to previously reported (Aston-Jones and Waterhouse, 2016), no AVP+ cell bodies were detected in the LC. This suggests that there was no local source of AVP within the LC and these AVP+ axons originate from other brain regions. AVP+ varicosities were also immunopositive for the vesicular glutamate transporter 2 (VGLUT2) (Figure 1B), a protein expressed exclusively in glutamatergic axon terminals. This suggests that AVP+ axons could form excitatory synapses with LC neurons.

To confirm this, we performed double peroxidase-chromogen immunostaining and TEM, using V – VIP (VECTOR^®^VIP Peroxidase (HRP) Substrate Kit; Vector Laboratories) and DAB as chromogens (see the “Materials and Methods” Section for details). It is worth noting that in EM preparations, the VIP reaction product was granular in appearance and easily distinguishable from the diffuse reaction product of DAB (Zhou and Grofova, 1995; Smiley et al., 1997; Muller et al., 2006, 2011). Visualization of the DAB reaction product at the light microscopical level revealed several AVP+ profiles (Figure 1C arrowheads and Figure 1D labeled as “AVP+ ax”). An AVP+ Herring body-like large varicose (Figure 1B white arrow and asterisk in Figure 1C,E), which is an anatomical feature of AVP-containing magnocell’s axons, was also evident (Figure 1C, asterisk). Within this field of view, two AVP+ bouton closely apposed to a TH+ dendrite (Figure 1C, boxed area). Examination of serial EM sections taken from the boxed area revealed that one of these AVP+ boutons formed an asymmetric (Gray type I) synapse with a TH+ dendrite (Figure 1C, insert). The presynaptic button contained abundant DAB precipitate and labeled dense core vesicles (dcv, AVP+, green arrowhead), characteristic of neuropeptide-containing axon terminals. Collectively, this demonstrates that AVP is contained in a set of LC afferents, which make excitatory synaptic connections with noradrenergic profiles of the LC.

### LC-NE Neurons Co-express mRNA for AVP V1a and V1b Receptors

**FIGURE 2:**
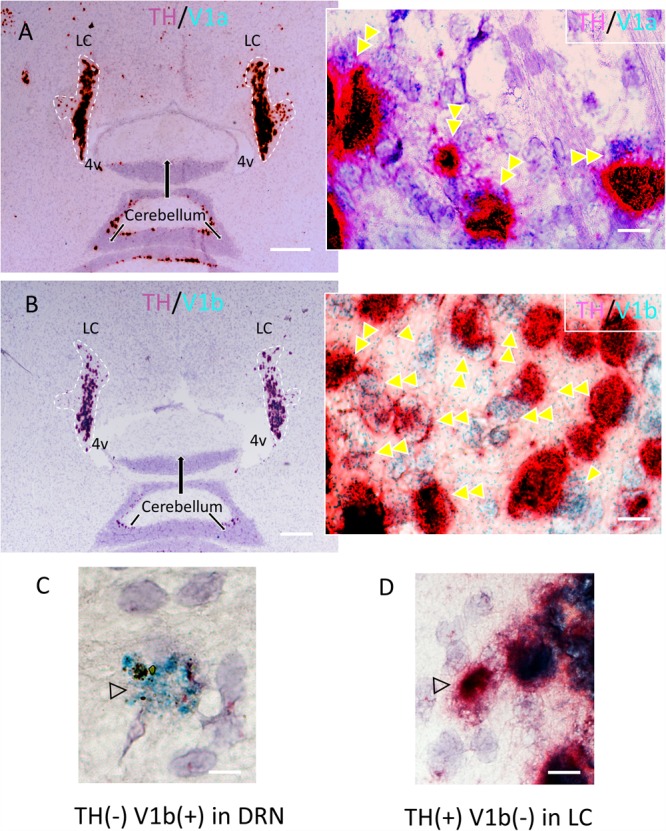
Confirmation of the expression of V1a and V1b receptor mRNA in noradrenergic LC neurons using a high resolution *in situ* hybridization method (RNAscope^®^2.5 HD Duplex Assay). **(A,B)** Horizontal sections of rat LC with inserts showing the co-localization (double arrowheads) of V1a/TH **(A)** and V1b/TH **(B)**. The TH mRNA signal was amplified using RNAscope channel 2 related probes and alkaline phosphatase (AP) – based Fast Red chromogen to result in a strongly detectable red signal and either V1a and V1b signals were amplified using RNAscope channel 1 related probes and horseradish peroxidase (HRP) – based green chromogen (the green punctuated signal). This newly developed technology allows a same-day ISH for two mRNAs detection with cellular resolution and being permanently mounted and examined under conventional light microcopy. Panels **(C,D)** are controls showing the labeling with V1b only, in the dorsal raphe nucleus and TH only in LC respectively. Scale bars: **A,B**: 0.5 mm; rest, 10 μm.

We recently demonstrated, using immunohistochemistry, that in the mouse, V1a and V1b, but not V2 receptors are expressed in the LC (Campos-Lira et al., 2018). Whilst the V1b was expressed by both noradrenergic and non-noradrenergic LC neurons, the V1a was exclusively expressed by LC noradrenergic neurons. We used a high resolution double *in situ* hybridization technique (RNAscope^®^2.5 HD Duplex Assay) to assess the comparative AVP receptor expression profile in rat LC. LC noradrenergic neurons, identified by mRNA signal for TH (Figure 2D) co-expressed signal for both the V1a (Figure 2A) and V1b (Figure 2B) receptors. This indicates that AVP, released at excitatory synapses, uses both V1a and V1b receptors for postsynaptic signaling within LC neurons. Figure 2C,D shows neurons located in the dorsal raphe nucleus and LC, respectively, which expressed either V1b mRNA but not TH mRNA (Figure 2C) or TH mRNA but not V1b mRNA (Figure 2D), from the same samples as experimental controls.

### AVP+ Axons in the LC Originate From the Magnocellular Neurosecretory Neurons of the PVN and SON

Our LM analysis revealed that the rat LC is devoid of AVP+ somata. This indicates that all AVP+ axons originate from regions beyond the LC. The presentation of LC AVP+ axons as large diameter profiles, with frequent-varicosities and Herring body-like structures which co-expressed the glutamatergic synaptic marker protein VGLUT2 is typical of hypothalamic AVPMNNs (Ziegler et al., 2002; Hrabovszky and Liposits, 2007), thus making this brain region the likely source of these afferents.

**FIGURE 3:**
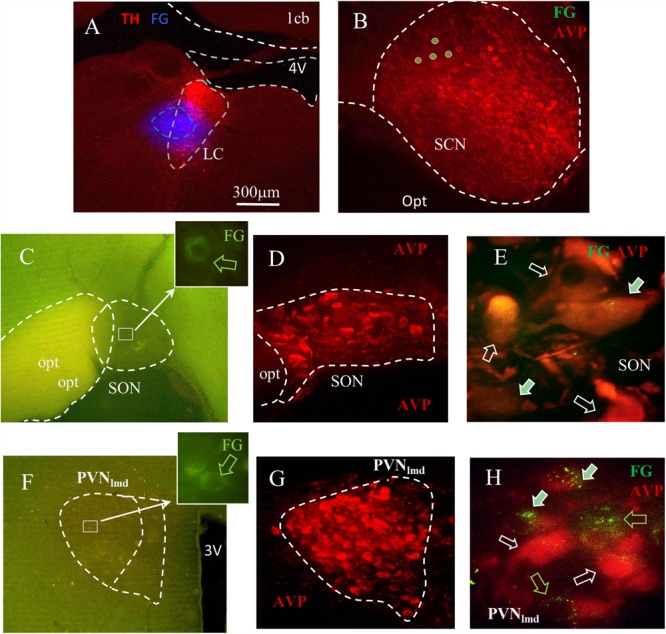
Fluoro-Gold (FG) retrograde tracing identifies the SON and PVN nuclei as the sources of LC AVP afferents. **(A)** Coronal section of pontine tegmentum, Bregma –9.84 mm; red TH+ IHC showing LC and blue FG labeling site of about 300 μm of diameter. **(B)** Within the suprachiasmatic nucleus (SCN), the FG labeled cells were found immuno-negative for AVP. In both SON **(C–E)** and the lateral magnocellular division of PVN (PVN_lmd_, **F–H**), around 20–60% of AVP+ neurons were positively labeled. **(C,F)** Photomicrographs from freshly vibratome sectioned coronal hypothalamic slices indicate the sparse FG labeled magnocellular neurons (perinuclear lysosome labeling pattern). FG and AVP immunohistochemistry were co-localized (filled arrows) in a population of the magnocellular neurons in SON **(D,E)** and PVN_lmd_ **(G,H)**. White hollow arrows indicate AVP+ cells without FG and green hollow arrows show the FG+ cells without AVP. See “Technical Considerations” Section in the “Discussion” for further considerations. Scale bars: 20 μm for **E,H**; rest 300 μm.

To assess this hypothesis, the retrograde tracer FG was stereotaxically injected in LC, and the transported signal then evaluated in target regions. A total of four animals were confirmed to have FG injections within the core of the LC, confirmed with TH IHC (see the “Materials and Methods” Section for detailed description) (Figure 3A). Inspection of AVP immunofluorescence in conjunction with FG signal, in regions of the anterior hypothalamus confirmed the co-expression of AVP-FG in the hypothalamic paraventricular (PVN) and supraoptic (SON) nuclei (Figure 3 panels C–H). This confirms that AVPMNNs provide AVP-immunopositive innervation of LC-NE neurons. Further interpretations and discussion of this finding are developed in the Section of “Technical Considerations” at the end of this paper.

Table 1 describes the semi-quantitative analysis of the four cases matching the inclusion criteria. Interestingly, AVP/FG double labeled neurons were not found inside the suprachiasmatic nucleus.

**Table 1:** Fluoro-Gold (FG) retrograde labeling experimental survey and anatomical description^∗^.

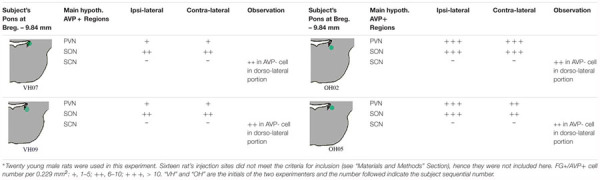

### Early Life Stress (ELS) Increases the Expression of AVP in the LC in Adulthood

**FIGURE 4:**
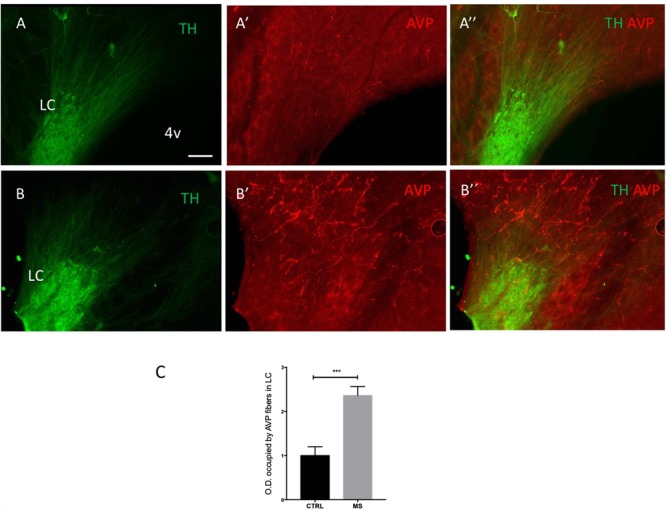
Early life stress in the form of neonatal maternal separation (MS) increases LC AVP immunoreactivity. Photomicrographs of rat brain horizontal sections (around dorso-ventral 7.30 mm), showing immunoreactivity for TH and AVP in the LC of control **(As)** and MS **(Bs)** subjects, treated as described in Materials and Methods, Maternal Separation (MS) Protocol. Note that all tissue was processed and imaged under identical conditions. **(C)** Quantification of AVP immunoreactivity. The columns represent the means and the errors represent the SEM; (*n* = 5); ^∗∗∗^*p* < 0.001. Scale bar: 100 μm.

We recently reported in mouse that acute stress significantly alters the expression of AVP terminals and AVP receptor expression in the mouse LC (Campos-Lira et al., 2018). The question therefore arises whether such stress-induced plasticity in the LC AVP system is enduring following exposure to chronic stress. One type of stress which induces long lasting molecular, physiological and structural plasticity in different brain regions (Caldji et al., 2003) including the LC (Swinny et al., 2010) is early life stress (ELS). Given the above data which indicate that LC AVP axons originate from, in part, the same center that integrates the stress response, i.e., the PVN, we assessed whether prior ELS alters the expression profile of AVP+ axons in the LC, using the maternal separation as a model of ELS. As previously reported for other brain regions (Zhang et al., 2012; Hernandez et al., 2016), MS resulted in a noticeable increase in the intensity of AVP immunoreactivity in the LC, in adulthood (Figure 4A,B). Quantification of AVP immunofluorescence intensity revealed a significant increase in MS samples (Figure 4C), *p* < 0.001, unpaired Student’s *t*-test; *n* = 5 animals.

### Water Deprivation 24 h (WD24h) Subtly Enhances Improvement in Spatial Learning and Memory Retention, Accompanied by a Significant Increase in Fos Expression in LC, Hippocampus and Prefrontal Cortex, After MWM Assessment

To answer our question whether the hypothalamic AVPMNN system’s sub-chronic upregulation exerts modulatory effects on the LC-NE neurons activation and subsequent projection regions, which could be reflected in their behavioral modifications, we devised an experiment to assess the spatial learning and memory retention in water-maze-naive rats using water deprivation (WD) as our experimental variable. From 12 h of water deprivation, the plasma AVP concentration in rat reaches to its maximum level and continues for the next 12 h (Zhang et al., 2010). Hence, this physiological model could up-regulate the AVP afference to LC.

**FIGURE 5:**
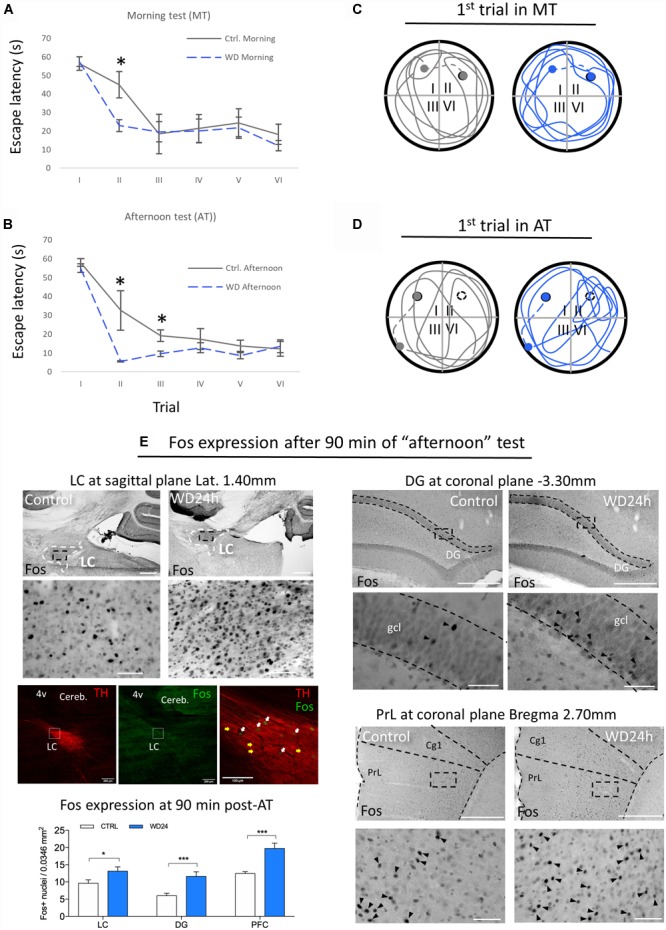
Morris water maze (MWM) test showed that WD24h improved spatial learning and memory retention as well as increasing neuronal activity in LC, hippocampus (assessed at suprapyramidal branch of granule cell layer (gcl) of dorsal dentate gyrus (DG) and prefrontal cortex [assessed at the layer 5 of prelimbic cortex (PrL), regions relevant for working memory]. **(A)** “Morning testing” (MT, see the “Materials and Methods” Section) showed that WD24h rats learned to locate the hidden platform (quadrant II) faster than control rats, as shown by a reduction in the latency to reach the platform in the second trial. **(B)** “Afternoon testing” (AT, see the “Materials and Methods” Section) confirmed the previous observation with a significant reduction of time to reach the escape-platform (quadrant I) in the second and third trials compared to control. **(C)** No apparent differences in the swimming strategy were seen as shown in a tracing of the swimming path of the first trial of MT. **(D)** A different swimming strategy was observed in the AT, with WD24h rats spending more time swimming in the quadrant where the platform was located (quadrant II) before finding the new location (quadrant I). In panels **(C,D)** filled circles symbolize the escape platform; hollow circles symbolize the old platform location; dots symbolize the rat location when 60 s exploration lapse ends; dashed lines symbolize the route that the experimenter guided the subject to the platform. **(E)** Photomicrographs and bar graphs show an increase in the number of Fos+ nuclei in the LC, granule cell layer (gcl) of dorsal dentate gyrus (DG), and prefrontal cortex assessed at the prelimbic cortex (PrL, layer 5), 90 min post the AT. Immuno-fluorescence images from WD subject’s LC (sagittal section) depict examples of Fos activation in LC-NE neurons (white arrows) after the MWM test. Note that some LC-NE neurons are not activated (yellow arrows). Scale bars 500 μm for the low amplifications photomicrographs and 50 μm for the high amplification; ^∗^*p* < 0.05; ^∗∗∗^*p* < 0.001.

As shown in Figure 5A, in the morning test, the WD group already showed a subtle improvement of spatial learning with a smoother learning course and a significant reduction of time to reach the escape-platform in the 2nd trial compared to control. This improvement is further confirmed in the afternoon session with significant reduction of time to reach the escape-platform in the 2nd and 3rd trials compared to control (Figure 5B). An interesting phenomenon was observed in the first trial of the afternoon test, in which the WD subjects spent more time swimming in the quadrant II where the escape-platform was located in the morning test, while the control rats did not show this preference (Figure 5C,D).

The expression of c-Fos, a marker of neuronal plasticity, assessed 90 min after the end point of MWM, revealed significant increase in the LC, as well as in the granule cell layer (gcl) of the dentate gyrus (DG) of dorsal hippocampus and prefrontal cortex. The panel E of Figure 5 shows a higher number of Fos+ nuclei in all regions evaluated comparing control vs. WD24h rats, i.e., LC: 9.62 ± 0.9 vs. 13.14 ± 1.2, *p* < 0.05; DG: 6.0 ± 0.7 vs. 11.62 ± 1.3, *p* < 0.001; and PFC: 12.45 ± 0.5 vs. 19.73 ± 1.5, *p* < 0.001. It is worth noting that both of the latter regions receive abundant NE innervations from LC (Schwarz et al., 2015) but no AVP direct innervations have been reported (Rood and De Vries, 2011; Zhang and Hernandez, 2013). Hence, it is coherent to interpret that these c-Fos expression increases are secondary to the potentiation of LC-NE afference to those regions.

## Discussion

In the current study, we provide the first demonstration that AVP-containing axons make synaptic connections with LC neurons. Furthermore, we provide evidence that these AVP afferents originate from magnocellular neurosecretory neurons (AVPMNNs) of the hypothalamic PVN and SON. Finally, our data suggest the recruitment of this AVPMNN circuit during periods of psychological and physiological challenges. Collectively, the study identifies the anatomical substrates linking two different neural systems with a common responsibility for ensuring homeostasis in complex environments.

### The Molecular and Structural Components of AVP-LC Communication

In contrast to its well-characterized role as a hormone in the periphery, within the brain, AVP has been shown to also exhibit the features of a neuromodulator by directly altering neuronal excitability (Zhang and Eiden, 2018). Axons containing such neuropeptide modulators generally adopt a range of release mechanisms, thereby increasing the versatility of their communication patterns. For example, volume transmission (VT) is a key feature of such hypothalamic neuropeptide neurotransmitters and facilitates the modulation of large ensembles of neurons (Alpar et al., 2018). The sparsity of AVP+ axons throughout the LC, in comparison to the vast expression of AVP receptors in virtually all LC neurons, suggests that the LC AVP system relies heavily on VT as a means of influencing LC neuronal excitability. However, our ultrastructural data indicate that AVP+ axons also employ the wired form of transmission in terms of forming conventional synapses with LC profiles. This suggests that AVP afferents impart highly directed, synaptic modulation of a sub-population of LC neurons, whilst maintaining a more general influence over the entire nucleus via VT. Since synaptic connectivity imparts speed and precision in neuronal network signaling, this raises the question whether the synaptically connected AVP-LC neurons represent a group of cells essential for mediating specific aspects of AVP-mediated brain functions, in a strict temporally constrained manner. This could result in the parcellation of the LC into different populations of neurons within AVP-LC pathways. Evidence for this heterogeneity of the LC AVP system stems from our recent report which demonstrated that the pharmacological activation of LC neurons resulted in contrasting effects on spontaneous firing rates (Campos-Lira et al., 2018). If so, identifying the anatomical and molecular signatures of this sub-populations of LC neurons, together with the source/s of such synapse innervating AVP+ axons, will be the crucial in furthering our understanding of the contribution of neurochemical systems to overall LC function.

### The Source of LC AVP+ Afferents

There are at least five major brain AVP centers, and therefore likely sources of the AVP+ axons innervating LC neurons, namely, the PVN, suprachiasmatic nucleus, supraoptic nucleus, bed nucleus of the stria terminalis (BNST), and medial amygdala (Goodson and Bass, 2001). AVP+ inputs to the LC have been reported since the late 1970s in both the core and pericoeruleus sub-regions (Buijs, 1978). These axons were considered to originate from parvocellular vasopressinergic neurons located within the caudal paraventricular nucleus and the BNST (Urban et al., 1999). However, the retrograde tracing data in this current studies point to magnocellular neurons of the PVN (AVPMNNs) being the major source. Our ancillary molecular data, in the form of VGLUT2-AVP co-expression, a signature of AVPMNNs, further supports the notion that these axons originate from this region of the brain. Further arguments to support the notion that these axons most likely arise from the hypothalamus rather than from extra-hypothalamic AVP-positive neurons include (i) AVPMNNs are known to be glutamatergic (Ziegler et al., 2002; Hrabovszky and Liposits, 2007); (ii) most, if not all, of the AVP-parvocellular populations, in hypothalamic suprachiasmatic nucleus (SCN), central (CeA) and medial (MeA) amygdala, bed nucleus of stria terminalis, intra-amygdala (BNSTIA) and medial posterior internal (BNSTmpi) divisions have been found to express VGAT hence to be GABAergic (Zhang and Eiden, unpublished; Zhang and Eiden, 2018). As such, the data reveal a synaptically connected circuit between PVN AVP+ neurons and the LC. Future studies focused on dissecting the roles of other PVN-LC pathways, such as those using CRF as a neuromodulator, are crucial in gaining a composite view of the contributions of such circuits to brain functions mediated by these respective centers.

### The LC-AVP System and Its Role in the Stress Response

A common feature of the LC and the PVN nuclei are their involvement in ensuring that the individual is capable of mediating a coordinated response to a variety of psychological and physiological stressors (Atzori et al., 2016). CRF has historically been considered the messenger of choice for both brain regions (Swinny and Valentino, 2006; Swinny et al., 2010). The current study, as well as our previous report, identifies AVP, within the LC, as an additional molecule in the arsenal of mediators used by these brain regions in times of stress. Our recent report showed that acute stress, in the form of restraint for 1 h, significantly increased the density of V1b receptors, while decreasing the density of AVP immunoreactivity. V1a receptors were unaffected (Campos-Lira et al., 2018). In the current study, we show that a more severe form of stress, namely MS, has the opposite effect on AVP expression, by significantly increasing the density of immunoreactive axons in the LC. Furthermore, a mild stress which is known to engage the PVN-AVP system, namely WD, together with novelty of being subjected to the MWM test, also appeared to engage the LC (in addition to other brain regions), based on the expression of the early gene marker Fos (Figure 5). Therefore, different life experiences have contrasting effects on LC-AVP plasticity. This stress-induced plasticity of the AVP in the brain has been replicated in other brain regions. For example, MS has been shown to potentiate the hypothalamic AVPMNN system as well as increase the fiber density in amygdala resulting in stress hyper-responsivity such as hyper-anxiety tested by Vogel-conflict test and elevated plus maze (Zhang et al., 2012; Hernandez et al., 2016). Remarkably, post-mortem analysis of the brains of suicide victims has indicated an increase in the immunoreactivity for AVP in the LC, in addition to other brain regions important in regulating emotions (Merali et al., 2006). Therefore, targeting the AVPMNNs system and the LC, in mental illnesses could unearth novel therapeutic strategies for debilitating stress-induced mental illnesses which remain poorly treated with currently available drug options.

### Does the Activation of This Pathway by 24 h Water Deprivation Lead to Enhancement of Memory and/or Learning?

Using MWM, we assessed spatial learning in rats after undergoing 24 h water-deprivation (WD), during which time the AVPMNNs are physiologically hyper-activated with only minimal changes in blood osmolality (Dunn et al., 1973; Zhang et al., 2010). We found here the memory retention and learning course were both improved in WD subject, while Fos-expression in LC, as well as in LC-NE projecting regions relevant for working memory, dorsal hippocampus and prefrontal cortex, were significantly increased. NE facilitates synaptic plasticity by recruiting and modifying multiple molecular elements of synaptic signaling, including specific transmitter receptors, intracellular protein kinases, and translation initiation have been extensively reported (Maity et al., 2015; Nguyen and Gelinas, 2018).

These results, adding to our previous studies, suggest that the AVPMNN system, a water homeostasis regulator, works centrally modulating the adaptive responses.

The postulated function of AVP as a modulator of LC principal neuron activity in response to osmotic stress is further supported by the finding of V1a receptors on TH+ (principal) LC neurons. Consistent with a role for AVP in linking osmotic stress to LC function, is enhancement of Fos up-regulation in LC neurons during a learning/orientation task (the MWM) after 24 h of water deprivation (WD) a reliable maneuver for enhancing AVPMNN tone in the PVN, SON and their vasopressinergic projections. In fact, performance during the MWM is also enhanced by WD. Since Fos activity is also up-regulated during MWM learning in LC target areas of the brain, some of which also receive AVPMNN inputs, it is not possible to know at this time if the effects of WD on MWM performance, though likely mediated through AVPMNN activity, are indirect (via action at the LC) or direct (via AVP release directly in these LC projection areas). Nevertheless, our findings reveal a novel neuroanatomical substrate for AVPMNN direct influence over LC neuronal function, and a new avenue for exploration of both homeostatic as well as allostatic modulation of behavior via the LC.

We take the data as a whole as indicating that the AVPMNN projections to LC likely act to potentiate a pattern of LC neuronal activity consistent with increased learning or enhanced attention under conditions of physiological stress (WD24h). This suggests that physiological need/motivation/stress enhances attention and learning under conditions in which acquisition of new information is especially critical to survival (Glennon et al., 2018).

### Technical Considerations: Limitations for Quantitative Conclusions

This study describes for the first time that the hypothalamic AVPMNNs innervate the LC using FG neurotracing, immunohistochemical, and neuroanatomical methods. FG injection into the LC revealed retrogradely labeled AVP-positive cells in hypothalamic supraoptic (SON) and paraventricular nuclei (PVN). There are several technical issues concerning this observation that we considered important to discuss here. The LC are bilateral dense groups of cells located in the pontine tegmentum, specifically in the lateral-rostral part of the floor of the 4th ventricle. Here, there are two technical challenges for neuro-circuit tracing. First, the rat LC is a relatively small nucleus (for instance, dimensions measured from confocal microcopy images from one rat were rostro-caudal 566 μm, dorso-ventral 306 μm, medio-lateral 320 μm). Second, it is located just below the 4th ventricle, thus it is extremely easy to have the tracer leaking into the cerebro-spinal fluid (CSF). Due the AVPMNNs neuro-secretion functional capacity, any leakage of FG can result in variable degree of magnocellular labeling, bilaterally. To avoid this leaking risk, we aimed to inject the tracer into the latero-ventral site of the LC as well as to use the method described in “Materials and Methods” which should yield a labeling region of less than 300 μm of diameter. In 20 attempts (20 rats used), only 4 resulted matching our inclusion criteria (20% of success rate). Although the stereotaxic coordinates and the rat body weights were kept identical, to the best of our knowledge, the first anatomical assessments for each rat yielded rather variable labeling sites. A more than 30 μm deviation, >10% of LC’s diameter, resulted in other structure’s labeling, which did not provide valid data for this study’s aims and were excluded for further analysis. The four successful cases were analyzed and reported in the Table 1 where we aimed to be rigorous and quantitative providing objective description of those four cases. We are fully aware that each injection only cover a small fraction of the LC’s dendritic field, in a variable degree. Hence, not global quantitative conclusion should be generated from those individual descriptions of the 4 rats. The value of this discovery is that only a small portion of the AVPMNNs were labeled resulting from a given local-injection of one region of LC, and we showed AVPMNNs without FG labeling but adjacent to the double labeled cells, which suggests that the labeling was not likely, at least part of them, to be through the humoral leakage. Hence, it demonstrates the existence of a monosynaptic pathway from AVNMNN to LC.

In summary, establishment of a linkage between the AVPMNN system and the LC adds a significant component to the already-established communication to the LC from the hypothalamus, via CRF projections from the PVN. That thirst enhances performance in the MWM concomitantly with enhanced activation of LC neurons during the conduct of the MWM test may indicate either enhancement of innate learning, or increased attention to cues presented during this test, and this too is an important new avenue that we intend to develop in the future. The resolution of this question will be helpful in determining the relative functional impact of increased vasopressinergic tone in LC compared to other brain regions which are supplied with AVPMNN afferents from the PVN and SON.

## Author Contributions

LZ conceived and led the study. VH, AN-K, OH-P, and LZ designed the experiments. OH-P, VH, AN-K, MS, and LZ performed the experiments. All authors analyzed the data. LZ, JS, RB, and LE contributed experimental reagents and resources. LZ wrote the manuscript with contribution of JS and LE, and feedback from the remaining authors.

## Conflict of Interest Statement

The authors declare that the research was conducted in the absence of any commercial or financial relationships that could be construed as a potential conflict of interest.

## References

[B1] Alpar A. Benevento M. Romanov R. A. Hokfelt T. Harkany T. (2018). Hypothalamic cell diversity: non-neuronal codes for long-distance volume transmission by neuropeptides. *Curr. Opin. Neurobiol.* 56 16–23. 10.1016/j.conb.2018.10.012 30471413

[B2] Alstein M. Whitnall M. H. House S. Key S. Gainer H. (1988). An immunochemical analysis of oxytocin and vasopressin prohormone processing in vivo. *Peptides* 9 87–105. 10.1016/0196-9781(88)90014-9 3362746

[B3] Armstrong W. (2004). “Hypothalamic supraoptic and paraventricular nuclei,” in *The Rat Nervous System* ed. Paxinos G. (Amsterdam: Elsevier) 369–388. 10.1016/B978-012547638-6/50016-X

[B4] Aston-Jones G. Waterhouse B. (2016). Locus coeruleus: from global projection system to adaptive regulation of behavior. *Brain Res.* 1645 75–78. 10.1016/j.brainres.2016.03.001 26969408 PMC4969192

[B5] Atzori M. Cuevas-Olguin R. Esquivel-Rendon E. Garcia-Oscos F. Salgado-Delgado R. Saderi C. (2016). Locus ceruleus norepinephrine release: a central regulator of CNS spatio-temporal activation? *Front. Synaptic Neurosci.* 8:25. 10.3389/fnsyn.2016.00025 27616990 PMC4999448

[B6] Berridge C. W. Waterhouse B. D. (2003). The locus coeruleus-noradrenergic system: modulation of behavioral state and state-dependent cognitive processes. *Brain Res. Brain Res. Rev.* 42 33–84. 10.1016/S0165-0173(03)00143-7 12668290

[B7] Buijs R. M. (1978). Intra- and extrahypothalamic vasopressin and oxytocin pathways in the rat. Pathways to the limbic system, medulla oblongata and spinal cord. *Cell Tissue Res.* 192 423–435. 10.1007/BF00224932 699026

[B8] Buijs R. M. Pool C. W. Van Heerikhuize J. J. Sluiter A. A. Van der Sluis P. J. Ramkema M. (1989). Antibodies to small transmitter molecules and peptides: production and application of antibodies to dopamine, serotonin, GABA, vasopressin, vasoactive intestinal peptide, neuropeptide y, somatostatin and substance P. *Biomed. Res.* 10(Suppl. 3) 213–221.

[B9] Caldji C. Diorio J. Meaney M. J. (2003). Variations in maternal care alter GABA(A) receptor subunit expression in brain regions associated with fear. *Neuropsychopharmacology* 28 1950–1959. 10.1038/sj.npp.1300237 12888776

[B10] Campos-Lira E. Kelly L. Seifi M. Jackson T. Giesecke T. Mutig K. (2018). Dynamic modulation of mouse locus coeruleus neurons by vasopressin 1a and 1b receptors. *Front. Neurosci.* 12:919. 10.3389/fnins.2018.00919 30618551 PMC6295453

[B11] Cui Z. Gerfen C. R. Young W. S. III (2013). Hypothalamic and other connections with dorsal CA2 area of the mouse hippocampus. *J. Comp. Neurol.* 521 1844–1866. 10.1002/cne.23263 23172108 PMC3798086

[B12] de Wied D. Diamant M. Fodor M. (1993). Central nervous system effects of the neurohypophyseal hormones and related peptides. *Front. Neuroendocrinol.* 14 251–302. 10.1006/frne.1993.1009 8258377

[B13] Dunn F. L. Brennan T. J. Nelson A. E. Robertson G. L. (1973). The role of blood osmolality and volume in regulating vasopressin secretion in the rat. *J. Clin. Invest.* 52 3212–3219. 10.1172/JCI107521 4750450 PMC302597

[B14] Fitzsimons J. T. O’Connor W. J. (1976). E. B. Verney’s demonstration of ’The antidiuretic hormone and the factors which determine its release’ [proceedings]. *J. Physiol.* 263 92–93. 796445

[B15] Glennon E. Carcea I. Martins A. R. O. Multani J. Shehu I. Svirsky M. A. (2018). Locus coeruleus activation accelerates perceptual learning. *Brain Res.* 10.1016/j.brainres.2018.05.048 [Epub ahead of print]. 29859972 PMC6274624

[B16] Goodson J. L. Bass A. H. (2001). Social behavior functions and related anatomical characteristics of vasotocin/vasopressin systems in vertebrates. *Brain Res. Brain Res. Rev.* 35 246–265. 10.1016/S0165-0173(01)00043-1 11423156

[B17] Hernandez V. S. Hernandez O. R. Perez de la Mora M. Gomora M. J. Fuxe K. Eiden L. E. (2016). Hypothalamic vasopressinergic projections innervate central amygdala GABAergic neurons: implications for anxiety and stress coping. *Front. Neural Circuits* 10:92. 10.3389/fncir.2016.00092 27932956 PMC5122712

[B18] Hernandez V. S. Ruiz-Velazco S. Zhang L. (2012). Differential effects of osmotic and SSR149415 challenges in maternally separated and control rats: the role of vasopressin on spatial learning. *Neurosci. Lett.* 528 143–147. 10.1016/j.neulet.2012.09.002 22982556

[B19] Hernandez V. S. Vazquez-Juarez H. Marquez E. Jauregui M. M. Huerta F. Barrio R. A. (2015). Extra-neurohypophyseal axonal projections from individual vasopressin-containing magnocellular neurons in rat hypothalamus. *Front. Neuroanat.* 9:130. 10.3389/fnana.2015.00130 26500509 PMC4593857

[B20] Hrabovszky E. Liposits Z. (2007). Glutamatergic phenotype of hypothalamic neurosecretory systems: a novel aspect of central neuroendocrine regulation. *Ideggyogy. Sz.* 60 182–186. 17451065

[B21] Kobayashi R. M. Palkovits M. Kopin I. J. Jacobowitz D. M. (1974). Biochemical mapping of noradrenergic nerves arising from the rat locus coeruleus. *Brain Res.* 77 269–279. 10.1016/0006-8993(74)90790-2 4152793

[B22] Kovacs K. J. (1998). c-Fos as a transcription factor: a stressful (re)view from a functional map. *Neurochem. Int.* 33 287–297. 10.1016/S0197-0186(98)00023-0 9840219

[B23] Kovacs K. J. (2008). Measurement of immediate-early gene activation- c-fos and beyond. *J. Neuroendocrinol.* 20 665–672. 10.1111/j.1365-2826.2008.01734.x 18601687

[B24] Landgraf R. Neumann I. D. (2004). Vasopressin and oxytocin release within the brain: a dynamic concept of multiple and variable modes of neuropeptide communication. *Front. Neuroendocrinol.* 25 150–176. 10.1016/j.yfrne.2004.05.001 15589267

[B25] Leng G. Dyball R. E. Luckman S. M. (1992). Mechanisms of vasopressin secretion. *Horm. Res.* 37 33–38. 10.1159/000182278 1398474

[B26] Levitt P. Moore R. Y. (1979). Origin and organization of brainstem catecholamine innervation in the rat. *J. Comp. Neurol.* 186 505–528. 10.1002/cne.901860402 15116686

[B27] Ludwig M. Leng G. (2006). Dendritic peptide release and peptide-dependent behaviours. *Nat. Rev. Neurosci.* 7 126–136. 10.1038/nrn1845 16429122

[B28] Maity S. Rah S. Sonenberg N. Gkogkas C. G. Nguyen P. V. (2015). Norepinephrine triggers metaplasticity of LTP by increasing translation of specific mRNAs. *Learn. Mem.* 22 499–508. 10.1101/lm.039222.115 26373828 PMC4579357

[B29] Merali Z. Kent P. Du L. Hrdina P. Palkovits M. Faludi G. (2006). Corticotropin-releasing hormone, arginine vasopressin, gastrin-releasing peptide, and neuromedin B alterations in stress-relevant brain regions of suicides and control subjects. *Biol. Psychiatry* 59 594–602. 10.1016/j.biopsych.2005.08.008 16197926

[B30] Morales M. Wang S. D. (2002). Differential composition of 5-hydroxytryptamine3 receptors synthesized in the rat CNS and peripheral nervous system. *J. Neurosci.* 22 6732–6741. 10.1523/JNEUROSCI.22-15-06732.2002 12151552 PMC6758137

[B31] Morris R. (1984). Developments of a water-maze procedure for studying spatial learning in the rat. *J. Neurosci. Methods* 11 47–60. 10.1016/0165-0270(84)90007-4 6471907

[B32] Muller J. F. Mascagni F. McDonald A. J. (2006). Pyramidal cells of the rat basolateral amygdala: synaptology and innervation by parvalbumin-immunoreactive interneurons. *J. Comp. Neurol.* 494 635–650. 10.1002/cne.20832 16374802 PMC2562221

[B33] Muller J. F. Mascagni F. McDonald A. J. (2011). Cholinergic innervation of pyramidal cells and parvalbumin-immunoreactive interneurons in the rat basolateral amygdala. *J. Comp. Neurol.* 519 790–805. 10.1002/cne.22550 21246555 PMC4586025

[B34] Nguyen P. V. Gelinas J. N. (2018). Noradrenergic gating of long-lasting synaptic potentiation in the hippocampus: from neurobiology to translational biomedicine. *J. Neurogenet.* 32 171–182. 10.1080/01677063.2018.1497630 30175650

[B35] Palkovits M. Brownstein M. J. (1983). “Locus coeruleus,” in *Advances in Cellular Neurobiology* Vol. 4 eds Fedoroff S. Hertz L. (Amsterdam: Elsevier) 81–103. 10.1016/B978-0-12-008304-6.50008-7

[B36] Robertson G. L. Shelton R. L. Athar S. (1976). The osmoregulation of vasopressin. *Kidney Int.* 10 25–37. 10.1038/ki.1976.76 181630

[B37] Robertson S. D. Plummer N. W. de Marchena J. Jensen P. (2013). Developmental origins of central norepinephrine neuron diversity. *Nat. Neurosci.* 16 1016–1023. 10.1038/nn.3458 23852112 PMC4319358

[B38] Rood B. D. De Vries G. J. (2011). Vasopressin innervation of the mouse (*Mus musculus*) brain and spinal cord. *J. Comp. Neurol.* 519 2434–2474. 10.1002/cne.22635 21456024 PMC3939019

[B39] Schmued L. C. Fallon J. H. (1986). Fluoro-Gold: a new fluorescent retrograde axonal tracer with numerous unique properties. *Brain Res.* 377 147–154. 10.1016/0006-8993(86)91199-6 2425899

[B40] Schwarz L. A. Luo L. (2015). Organization of the locus coeruleus-norepinephrine system. *Curr. Biol.* 25 R1051–R1056. 10.1016/j.cub.2015.09.039 26528750

[B41] Schwarz L. A. Miyamichi K. Gao X. J. Beier K. T. Weissbourd B. DeLoach K. E. (2015). Viral-genetic tracing of the input-output organization of a central noradrenaline circuit. *Nature* 524 88–92. 10.1038/nature14600 26131933 PMC4587569

[B42] Smiley J. F. Morrell F. Mesulam M. M. (1997). Cholinergic synapses in human cerebral cortex: an ultrastructural study in serial sections. *Exp. Neurol.* 144 361–368. 10.1006/exnr.1997.6413 9168836

[B43] Swanson L. W. (1976). The locus coeruleus: a cytoarchitectonic, Golgi and immunohistochemical study in the albino rat. *Brain Res.* 110 39–56. 10.1016/0006-8993(76)90207-9 776360

[B44] Swinny J. D. O’Farrell E. Bingham B. C. Piel D. A. Valentino R. J. Beck S. G. (2010). Neonatal rearing conditions distinctly shape locus coeruleus neuronal activity, dendritic arborization, and sensitivity to corticotrophin-releasing factor. *Int. J. Neuropsychopharmacol.* 13 515–525. 10.1017/S146114570999037X 19653930 PMC2857591

[B45] Swinny J. D. Valentino R. J. (2006). Corticotropin-releasing factor promotes growth of brain norepinephrine neuronal processes through Rho GTPase regulators of the actin cytoskeleton in rat. *Eur. J. Neurosci.* 24 2481–2490. 10.1111/j.1460-9568.2006.05129.x 17100837

[B46] Urban I. J. A. Burbach J. P. H. De Wied D. (1999). *Advances in Brain Vasopressin.* Amsterdam: Elsevier.

[B47] Valentino R. J. Van Bockstaele E. (2008). Convergent regulation of locus coeruleus activity as an adaptive response to stress. *Eur. J. Pharmacol.* 583 194–203. 10.1016/j.ejphar.2007.11.062 18255055 PMC2349983

[B48] Verney E. B. (1947). The antidiuretic hormone and the factors which determine its release. *Proc. R. Soc. Lond. B Biol. Sci.* 135 25–106. 10.1098/rspb.1947.0037 18918876

[B49] Yamaguchi T. Wang H. L. Li X. Ng T. H. Morales M. (2011). Mesocorticolimbic glutamatergic pathway. *J. Neurosci.* 31 8476–8490. 10.1523/JNEUROSCI.1598-11.2011 21653852 PMC6623324

[B50] Ying C. Qi Y. Cang-Bao X. (2017). A convenient method for quantifying collagen fibers in atherosclerotic lesions by ImageJ software. *Int. J. Clin. Exp. Med.* 10 14904–14910.

[B51] Zhang L. Eiden L. E. (2018). Two ancient neuropeptides, PACAP and AVP, modulate motivated behavior at synapses in the extrahypothalamic brain: a study in contrast. *Cell Tissue Res.* 375 103–122. 10.1007/s00441-018-2958-z 30519837

[B52] Zhang L. Hernandez V. S. (2013). Synaptic innervation to rat hippocampus by vasopressin-immuno-positive fibres from the hypothalamic supraoptic and paraventricular nuclei. *Neuroscience* 228 139–162. 10.1016/j.neuroscience.2012.10.010 23085097

[B53] Zhang L. Hernandez V. S. Liu B. Medina M. P. Nava-Kopp A. T. Irles C. (2012). Hypothalamic vasopressin system regulation by maternal separation: its impact on anxiety in rats. *Neuroscience* 215 135–148. 10.1016/j.neuroscience.2012.03.046 22522466

[B54] Zhang L. Hernández V. S. Medina-Pizarro M. Valle-Leija P. Vega-González A. Morales T. (2008). Maternal hyperthyroidism in rats impairs stress coping of adult offspring. *J. Neurosci. Res.* 86 1306–1315. 10.1002/jnr.21580 18074386

[B55] Zhang L. Hernandez V. S. Swinny J. D. Verma A. K. Giesecke T. Emery A. C. (2018). A GABAergic cell type in the lateral habenula links hypothalamic homeostatic and midbrain motivation circuits with sex steroid signaling. *Transl. Psychiatry* 8:50. 10.1038/s41398-018-0099-5 29479060 PMC5865187

[B56] Zhang L. Hernandez V. S. Vazquez-Juarez E. Chay F. K. Barrio R. A. (2016). Thirst is associated with suppression of habenula output and active stress coping: is there a role for a non-canonical vasopressin-glutamate pathway? *Front. Neural Circuits* 10:13. 10.3389/fncir.2016.00013 27065810 PMC4814529

[B57] Zhang L. Medina M. P. Hernandez V. S. Estrada F. S. Vega-Gonzalez A. (2010). Vasopressinergic network abnormalities potentiate conditioned anxious state of rats subjected to maternal hyperthyroidism. *Neuroscience* 168 416–428. 10.1016/j.neuroscience.2010.03.059 20371268

[B58] Zhou M. Grofova I. (1995). The use of peroxidase substrate Vector VIP in electron microscopic single and double antigen localization. *J. Neurosci. Methods* 62 149–158. 10.1016/0165-0270(95)00069-0 8750097

[B59] Ziegler D. R. Cullinan W. E. Herman J. P. (2002). Distribution of vesicular glutamate transporter mRNA in rat hypothalamus. *J. Comp. Neurol.* 448 217–229. 10.1002/cne.10257 12115705

